# The Geriatric Nutritional Risk Index Independently Predicts Mortality in Diabetic Foot Ulcers Patients Undergoing Amputations

**DOI:** 10.1155/2017/5797194

**Published:** 2017-01-09

**Authors:** Yuanyuan Xie, Hailing Zhang, Tingting Ye, Shengjie Ge, Ruyi Zhuo, Hong Zhu

**Affiliations:** Department of Endocrinology and Metabolism, The First Affiliated Hospital of Wenzhou Medical University, Wenzhou, China

## Abstract

*Objective*. Patients with diabetic foot ulcers undergoing amputations have poor prognosis. Malnutrition usually occurs in this population and is associated with increased risk of mortality. The geriatric nutritional risk index (GNRI) is a widely used, simple, and well-established tool to assess nutritional risk. The purpose of this study was to assess the association between GNRI and all-cause mortality in diabetic foot ulcers patients undergoing minor or major amputations.* Methods*. This was a retrospective cohort study including 271 adult patients. Patients were divided into two groups according to a GNRI cutoff value of 92, and characteristics and mortality were compared between the two groups. Cox proportional hazard analysis was performed to explore the association between GNRI and mortality.* Result*. GNRI (*p* < 0.001), age (*p* < 0.001), and eGFR (*p* = 0.002) were independent predictors of mortality. Among a subgroup of 230 patients with minor amputation, increased age (*p* < 0.001), coronary artery disease (*p* = 0.030), and increased GNRI (*p* < 0.001) were major risk factors.* Conclusion*. GNRI on admission might be a novel clinical predictor for the incidence of death in patients with diabetic foot ulcers who were undergoing amputations.

## 1. Introduction

The prevalence of type 2 diabetes has been increasing rapidly throughout the world. In 2015, the International Diabetes Federation estimated that 1 in 11 adults aged between 20 and 79 years had diabetes and that the number of adults worldwide living with diabetes had soared to 425 million. This number is projected to increase to 642 million by 2040, with a global prevalence of 10.0% [1]. Diabetic foot ulcers (DFU) are a common complication in diabetic patients, with the lifetime incidence of developing a foot ulcer estimated to be up to 25% [2]; moreover, DFU are the leading cause of nontraumatic lower extremity amputation (LEA) worldwide [3]. Furthermore, LEA is related to a high risk of death in diabetic subjects [4, 5]. Diabetic patients undergoing LEA have increased risk of mortality compared with those who have not undergone LEA [6].

A high prevalence of malnutrition, which is frequently unrecognized among patients with chronic or severe diseases, is significantly associated with a longer hospital stay as well as increased morbidity and mortality [7, 8]. Nutritional deficiencies and poor food intake play a major role in the impaired nutritional status of patients with diabetes-related complications, including renal failure and foot infection [9]. DFU patients, who have a high occurrence of infection and vascular complications, more commonly suffer from malnutrition compared with patients without DFU [10].

A geriatric nutritional risk index (GNRI) has been used as a simple and valuable tool to predict outcomes calculated from only serum albumin and the ratio between actual and ideal body weight [11]. GNRI is a prognostic determinant for clinical outcomes in patients with chronic heart failure [12] and those on hemodialysis [13]. In addition, GNRI has been used as a predictor of amputation in patients with critical limb ischemia [14, 15]. However, GNRI is rarely adopted to evaluate the nutritional condition of patients suffering from DFU. Moreover, the value of GNRI for predicting mortality in DFU patients who have experienced or are undergoing LEA has not been explored.

Therefore, the aim of the present study was to determine the predictive relationship between GNRI and prognosis among DFU patients undergoing LEA.

## 2. Materials and Methods

### 2.1. Study Population

This was a retrospective cohort study including 271 adult patients with diagnoses of type 2 diabetes mellitus and diabetic foot, who had consented to receive minor or major amputation at The First Affiliated Hospital of Wenzhou Medical University between May 2010 and May 2015. Minor amputation was defined as any amputation distal to the ankle joint, and major amputation was defined as amputation above the ankle. The diagnosis of diabetes mellitus was based on World Health Organization criteria or on having been treated with insulin and/or oral hypoglycemic agents. Hypertension was diagnosed if one of the following conditions was present: systolic blood pressure ≥ 140 mmHg, diastolic blood pressure ≥ 90 mmHg, or the presence of treatment for hypertension. The coronary artery disease was defined by medical history and clinical symptoms of the coronary artery. The cerebral vascular disease was assumed to be present with any event of neurologic deficiency, whether persistent or resolved. Peripheral arterial disease was diagnosed by the presence of stenosis as shown in Doppler ultrasound performed by specialist physicians.

The present study was approved by the ethics committees and was conducted in accordance with Declaration of Helsinki.

### 2.2. Methods and Calculations

Demographic variables (age and sex), anthropometric parameters (height, weight, and body mass index [BMI]), medical history, history of smoking and alcohol abuse, and comorbidity and laboratory data (e.g., albumin, creatinine) were collected from individual medical records upon admission according to prespecified definitions. Body mass index was calculated as bodyweight divided by height squared (kg/m^2^). The estimated glomerular filtration rate (eGFR) was calculated according to the Chronic Kidney Disease Epidemiology Collaboration formula, and a physical examination was performed to determine Wagner grade of the foot lesion [16]. Follow-up data were obtained in medical records or by telephone interview. Mortality during follow-up was used as the outcome measure. 

*GNRI Calculation*. GNRI was calculated from individually obtained height in cm, current body weight in kg, ideal body weight, and serum albumin level as follows [11]:

(1) GNRI=1489×albuming/L+41.7×weightWLo,

where WLo indicates ideal weight, which was calculated as follows:

(2) $$\begin{array}{c} \text{men: }WLo=H-100-\left[\;\frac{\left(H-150\right)}{4}\;\right],\\ \text{women: }H-100-\left[\;\frac{\left(H-150\right)}{2.5}\;\right], \end{array}$$

where *H* indicates height.

From these GNRI values, four grades of nutrition-related risk were defined [11]: high risk (<82), moderate risk (82 to <92), low risk (92–98), and no risk (>98).

### 2.3. Statistical Analysis

Continuous variables are expressed as mean ± standard deviation (SD) for normally distributed variables. Variables exhibiting a nonnormal distribution are expressed as median and interquartile range. Categorical variables are expressed as percentages and were compared using the chi-square test. Differences in continuous variables were compared using Student's *t*-test for normally distributed variables and the Whitney *U* test for nonnormally distributed variables. Survival analysis was estimated using the Kaplan-Meier method, and differences were compared using the log-rank test. The independent association between GNRI and mortality was assessed using the Cox proportional hazard model, which was conducted using stepwise regression. Height, weight, serum albumin, and creatinine were excluded as they were used in the calculation of GNRI, BMI, and eGFR. All analyses were performed using IBM SPSS Statistics version 23. *p* < 0.05 was considered statistically significant.

## 3. Results

### 3.1. Baseline Demographic and Clinical Characteristics

**Figure 1:**
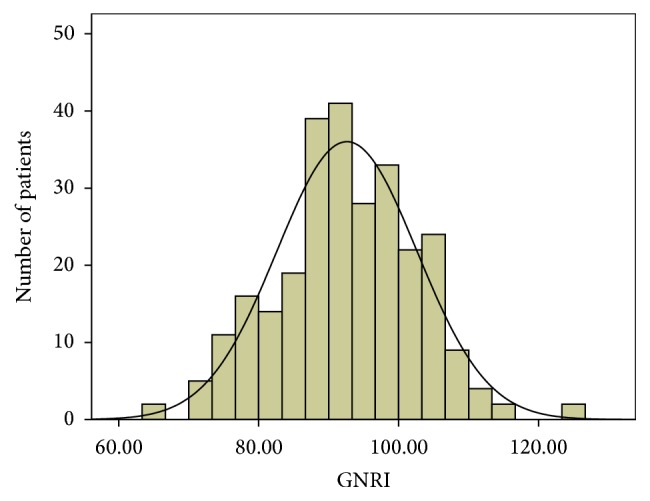
Distribution of geriatric nutritional risk index (GNRI).

Baseline clinical characteristics and laboratory data for all participants were listed in Table 1. Mean age was 66.9 ± 11.1 years; 59.8% of participants were men, 93.7% had peripheral arterial disease, 60.9% had hypertension, 55.9% had albuminuria, 14% had cerebral vascular disease, 9.6% had coronary artery disease, 19.2% had lower limb revascularization, 34.7% were smokers, and 29.2% were drinkers.  Figure 1 shows the distribution of GNRI in the DFU patients undergoing LEA in this study. Mean baseline GNRI was 92.61 ± 10.00. Patients were divided into two groups based on the following cutoff value: high GNRI (GNRI ≥ 92, *n* = 138) with low or no nutritional risk and low GNRI (GNRI < 92, *n* = 133) with moderate or severe risk.

### 3.2. Patient Characteristics according to GNRI

As shown in Table 1, the duration of diabetic foot ulcers was longer in the high GNRI group. In addition, BMI, systolic blood pressure, serum hemoglobin, albumin, cholesterol, and high-density lipoprotein (HDL-C) values were significantly higher in high GNRI group. Blood platelet counts, white blood cell counts, low-density lipoprotein (LDL-C) values, and fibrinogen levels were significantly higher in the low GNRI group. The prevalence of smoking was significantly higher in the high GNRI group, and the proportion of major amputation was significantly higher in the low GNRI group. There were no statistically significant between-group differences in eGFR; age; sex; comorbidity; history of lower limb revascularization; drinking; albuminuria; Wagner classification; lymphocyte, triglycerides, and creatinine; blood urea nitrogen, uric acid, fasting glucose levels, or systolic blood pressure.

### 3.3. Causes of Death

Of the 271 subjects who completed the follow-up, a total of 72 (26.6%) subjects died. The causes of death included the following: 22 (30.1%) foot-related deaths, 13 (18.0%) deaths due to cardiac disease, 10 (13.9%) deaths due to cerebrovascular disease, 9 (12.5%) deaths due to renal failure, 7 (9.7%) deaths due to infections, 2 (2.8%) deaths due to cancer-related death, 2 (2.8%) deaths due to acute complication of diabetes, and 7 (9.7%) deaths with other causes.

### 3.4. Predictors of Mortality after Amputation

**Figure 2:**
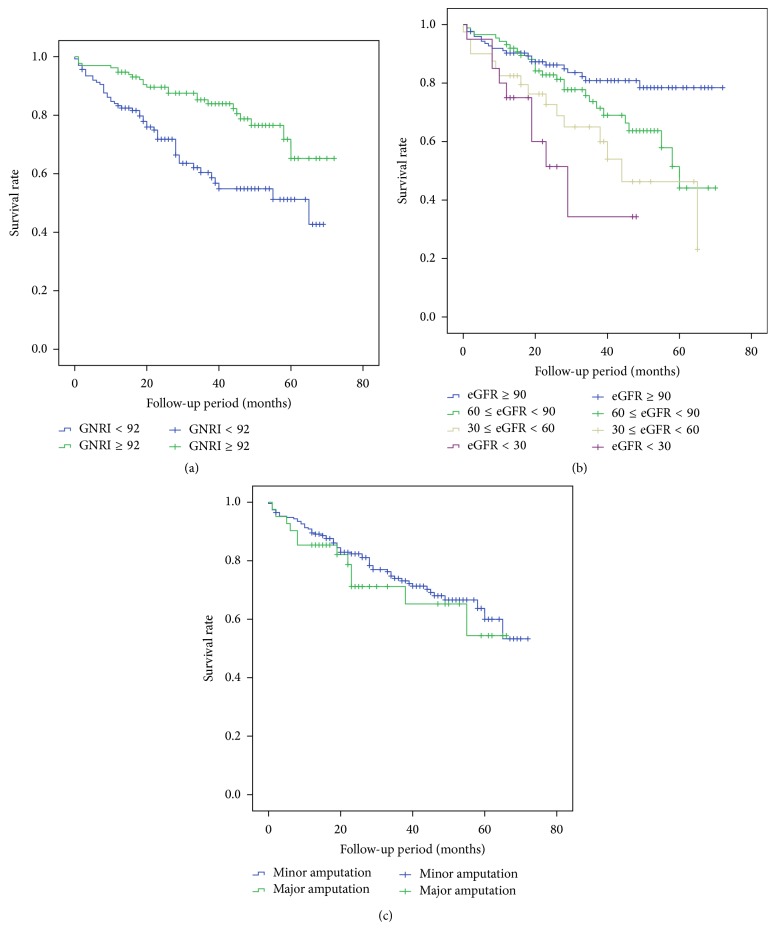
Kaplan-Meier survival curves of the proportion of patients with mortality, according to (a) GNRI categories (low GNRI, <92; high GNRI, ≥92), (b) eGFR stratification (eGFR, ≥90; 60 ≤ eGFR < 90; 30 ≤ eGFR < 60; eGFR < 30), or (c) amputation categories (minor amputation; major amputation). The differences between groups in (a) and (b) were significant (log-rank test, all *p* < 0.001). There was no significant difference in (c) between groups (log-rank test, *p* = 0.496).

Kaplan-Meier survival analysis for all-cause mortality is shown in Figure 2. The overall survival rate was significantly lower in the low GNRI group (log-rank *p* < 0.001). A significant increase in the incidence of death was observed in patients with increased eGFR (log-rank *p* < 0.001). Mortality rates at 1, 3, 5, and 6 years after amputation were 11%, 27%, 41%, and 47%, respectively. Mean survival times (months) in the low GNRI group were 45.797 ± 2.568 (95% CI 40.763–50.831) and in the high GNRI group were 60.084 ± 2.151 (95% CI 55.868–64.301).

As shown in Table 2, univariate analysis revealed that the following variables were significantly associated with mortality: age (hazard ratio 1.044, 95% CI 1.021–1.068, and *p* < 0.001), BMI (hazard ratio 0.918, 95% CI 0.850–0.991, and *p* = 0.029), hypertension (hazard ratio 1.867, 95% CI 1.121–3.109, and *p* = 0.016), hemoglobin (hazard ratio 0.985, 95% CI 0.972–0.998, and *p* = 0.028), albumin (hazard ratio 0.945, 95% CI 0.906–0.985, and *p* = 0.008), creatinine (hazard ratio 1.002, 95% CI 1.001–1.003, and *p* = 0.001), eGFR (hazard ratio 0.985, 95% CI 0.977–0.992, and *p* < 0.001), and GNRI (hazard ratio 0.960, 95% CI 0.938–0.983, and *p* = 0.001). In the multivariate Cox proportional hazard model analysis, GNRI as a continuous variable (hazard ratio 0.945, 95% CI 0.921–0.971, and *p* < 0.001) and age (hazard ratio 1.046, 95% CI 1.022–1.071, *p* < 0.001) and eGFR (hazard ratio 0.987, 95% CI 0.979–0.995, *p* = 0.002) were independent predictors of mortality.

### 3.5. Predictors of Mortality after Minor Amputation

As shown in Table 3, univariate analysis revealed significant association with mortality for the following variables: age (hazard ratio 1.053, 95% CI 1.027–1.079, and *p* < 0.001), cerebral vascular disease (hazard ratio 1.979, 95% CI 1.070–3.662, and *p* = 0.016), coronary artery disease (hazard ratio 2.286, 95% CI 1.119–4.671, and *p* = 0.023), creatinine (hazard ratio 1.002, 95% CI 1.001–1.004, and *p* = 0.008), eGFR (hazard ratio 0.986, 95% CI 0.977–0.994, and *p* = 0.001), and GNRI (hazard ratio 0.960, 95% CI 0.935–0.987, and *p* = 0.001). In the multivariate Cox proportional hazard model analysis, GNRI as a continuous variable (hazard ratio 0.936, 95% CI 0.908–0.965, and *p* < 0.001), age (hazard ratio 1.061, 95% CI 1.033–1.089, and *p* < 0.001), and coronary artery disease (hazard ratio 2.291, 95% CI 1.086–4.836, and *p* = 0.030) were independent predictors of mortality.

## 4. Discussion

In the present study, we investigated the validity of GNRI for predicting all-cause death among DFU patients who underwent LEA. To the best of our knowledge, this is the first study demonstrating that lower GNRI before surgery was significantly associated with higher mortality among these patients. As expected, DFU patients undergoing LEA in the current study had high mortality (mean survival 53.8 months). This agrees with data from previous reports of high mortality rates in diabetic patients with amputations [4, 17]. From the current and prior results, we propose using GNRI for nutritional evaluation to predict mortality and provide a basis for appropriate and timely nutritional treatment in DFU patients.

Previous studies have demonstrated that older age and a higher level of amputation have a worse prognosis than do minor amputees [18–20]. However, the study from Taiwan found that major amputation is not an associated risk factor for mortality after adjusting for sex and age [21]. Another study demonstrated that there is no difference in prognosis between major and minor amputations at two years if the surgical wounds could heal [22]. In our study, wounds healed after amputations among most patients, which might explain that the level of amputation was not associated with mortality. Furthermore, whether the surgical procedure of minor LEA was too conservative in some severe cases who should have had a higher level of amputation was not known. Our findings again stress the point that the coronary artery disease [22] and renal function [18] are associated with mortality in diabetic patients after lower extremity amputations.

Bouillanne et al. were the first to assess that GNRI was predictive of mortality in hospitalized elderly patients [11]. Subsequently, the predictive value of GNRI for prognosis has also been demonstrated for older age, dialysis patients, and cardiovascular patients [10–13]. The Mini-Nutritional Assessment (MNA) is a well-validated nutritional screening tool with high sensitivity and specificity and is widely used in clinical practice to measure nutritional status [23]. GNRI has a poorer tendency to classify patients as being at risk or malnourished than MNA but appears to better predict outcomes [24–27]. According to these studies, GNRI is not an index of malnutrition but is useful as a nutrition-related risk index based on GNRI cutoff values. Therefore, it is reasonable to consider that adoption of GNRI is appropriate for predicting risk of mortality and morbidity.

Malnutrition is common in patients with limb-threatening DFU and has a significant influence on prognosis [10], including delayed wound healing [28], longer hospital stay, and increased mortality [7, 8, 29]. Moreover, as nutritional status is related to immunity, patients with poor nutritional status more commonly suffer from infection [30]. The weakened immune system in these malnourished patients may help to explain the high rate of infection-related complications and mortality.

Foot infections are a consequence of foot ulceration and have the potential to expand and deepen, which can result in loss of limb [31]. Moreover, the inflammatory response to foot infection leads to high metabolism, which requires more nutrients and is associated with protein deficiency during the wound healing process [28]. Consistent with this, serum albumin level is lower in DFU patients who required amputation [32]. Mean serum albumin in the current study was 33.25 g/L, which is lower than normal serum concentration. In general, patients with more severe disease have a lower serum albumin concentration [33].

Serum albumin is considered as a clinical monitoring tool for nutritional assessment, and hypoalbuminemia is strongly correlated with complications and mortality in the elderly [34–36]. Therefore, hypoalbuminemia is considered as a predictive risk of mortality. With advancing age, serum albumin level gradually decreases [37], suggesting an impaired ability to adapt to disease-related metabolic stress in elderly persons [25]. The present results confirm that poor clinical outcome is associated with hypoalbuminemia in patients who may not have been able to cope with the metabolic stress of foot infection. Furthermore, albumin is an important factor in the GNRI formula and thus may potentially explain the relationship between GNRI and mortality among DFU patients undergoing LEA.

Clinically, pain from ulcers and surgery may prevent physical activity, resulting in disuse atrophy of skeletal muscle. In addition, lower dietary intake and increased nutrient needs, which paradoxically exist in association with diseases, lead to muscle catabolism with subsequent negative effects on muscle strength [38]. Furthermore, higher levels of inflammation markers, which are commonly found in diabetes [39], are associated with decreased muscle strength and function [40]. Therefore, diabetes accelerates the loss of muscle strength and quality [39]. In addition, there is also a clear inverse relationship between muscle strength and mortality [41]. Furthermore, GNRI is predictive of muscle dysfunction [42, 43]. Accordingly, the relationship between low GNRI and high mortality may involve impaired muscle strength.

A number of therapies are available to reduce ulcer risk, heal wounds, and prevent amputations [31, 44, 45]. Unfortunately, LEA is often performed as the ultimate endpoint of diabetic foot disease, and current treatments for these patients are not as effective as they should be. On the other hand, in patients after surgery or injury, the hypermetabolic response persists for a long time, leading to large losses of protein from skeletal muscle [46]. Based on protein intake from the typical three daily meals, patients may not consume sufficient nitrogen to meet their hypermetabolic need.

We suggest the necessity of appropriate and timely nutritional support to improve clinical outcomes, especially reduced mortality. There is evidence that such intervention may be successful. For example, in a group of patients at risk of malnutrition, losses in muscle strength and weight were prevented by nutritional supplementation [47].

Moreover, dynamic changes in serum albumin have been observed from admission to discharge [35]. Throughout hospitalization, hypoalbuminemia develops or is maintained if present on admission. Improvement of serum albumin from low to normal levels may help decrease mortality. Because nutritional status may be improved with appropriate interventions, this may help maintain muscular functional capacity, improve serum albumin level, and recover immune function, although additional studies are needed to verify this. We conclude that GNRI should be used to evaluate the appropriate nutritional intervention.

There are several limitations to the present study. First, it was a single-center study with a relatively small sample size; thus the results may not be representative for patients with diabetes-related amputations in general. Second, the GNRI values were not measured with a disease-specific scale, such as the diabetic foot disease scale, which would provide a more adequate measurement. Third, it remains unclear how high malnutrition risk reflects the prevalence of malnutrition. As mentioned above, GNRI is a nutrition-related risk index. Unfortunately, detailed nutritional and other information, such as dietary intake, weight loss, and muscle strength or mass, was not obtained in the current study. Furthermore, there is currently no gold standard for diagnosing malnutrition. Fourth, we only analyzed the predictive value of GNRI before surgery and did not analyze the impact of dynamic changes in GNRI on mortality. The present findings need to be confirmed by further studies, and future prospective studies with a larger sample size and a multicenter design should evaluate if disease-specific GNRI is associated with clinical outcomes.

In conclusion, GNRI on admission is an effective predictive marker for mortality in DFU patients undergoing LEA.

## Figures and Tables

**Table 1:** Baseline patient characteristics.

	Overall	High GNRI (≥92)	Low GNRI (<92)	*p* value
Case *n* (%)	271	133	138	

Age, years	66.9 ± 11.1	67.4 ± 11.4	66.4 ± 10.8	0.444

Male *n* (%)	162 (59.8)	83 (62.4)	79 (57.2)	0.387

BMI, kg/m^2^	23.0 ± 3.1	24.4 ± 2.9	21.7 ± 2.6	<0.001

Systolic blood pressure, mmHg	243.6 ± 23.0	147.5 ± 23.7	139.8 ± 21.8	0.006
Diastolic blood pressure, mmHg	75.0 ± 11.9	76.0 ± 11.7	74.0 ± 12.0	0.155

Duration of diabetes, years	10.0 (4.0–15.0)	10 (4.0–15.0)	10.0 (5–15.3)	0.197

Duration of diabetic foot ulcers, months	1.0 (0.67–3)	2 (0.8–4)	1 (0.5–3.0)	0.031

Wagner classification				0.073
2	14 (4.2)	7 (5.1)	7 (5.3)	
3	91 (33.6)	52 (39.1)	39 (28.3)	
4	162 (33.6)	74 (55.6)	88 (63.8)	
5	4 (1.5)	0 (0)	4 (2.9)	

Comorbidity *n* (%)				
Hypertension	165 (60.9)	88 (66.2)	77 (55.8)	0.080
Cerebral vascular disease	38 (14.0)	17 (12.8)	21 (15.2)	0.564
Coronary artery disease	26 (9.6)	17 (12.8)	9 (6.5)	0.080
Peripheral arterial disease	254 (93.7)	127 (95.5)	127 (92)	0.240

History *n* (%)				
Smoking (current or ever)	94 (34.7)	55 (41.4)	39 (28.6)	0.024
Drinking (current or ever)	79 (29.2)	42 (31.6)	37 (26.8)	0.388
lower limb revascularization	52 (19.2)	30 (22.6)	22 (15.9)	0.167

Laboratory date				
Hemoglobin, g/L	108.5 ± 18.6	113.5 ± 18.2	103.7 ± 17.8	<0.001
White blood cell, *∗*10^9^/L	8.9 (6.9–11.7)	8.23 (6.0–10.4)	10.0 (7.8–13.01)	<0.001
Lymphocyte, *∗*10^9^/L	1.5 ± 0.7	1.6 ± 0.7	1.5 ± 0.6	0.530
Blood platelet *∗*10^9^/L	276.6 ± 101.4	248 ± 85.0	303 ± 108.6	<0.001
Albumin, g/L	33.25 ± 5.5	36.9 ± 3.9	29.7 ± 4.4	<0.001
Cholesterol, mmol/L	4.2 ± 1.2	4.4 ± 1.1	4.0 ± 1.2	0.004
Triglycerides, mmol/L	1.24 (0.91–1.24)	1.3 (0.9–1.9)	1.2 (0.9–1.6)	0.273
HDL-C, mmol/L	0.9 ± 0.3	1.0 ± 0.3	0.8 ± 0.3	<0.001
LDL-C, mmol/L	2.4 (1.8–3.0)	2.3 (1.8–2.9)	2.5 (1.9–3.10)	0.025
Creatinine, *μ*mol/L	69 (56–92)	71 (57–92.5)	67.0 (54.0–91.5)	0.353
Blood urea nitrogen, mmol/L	5.8 (4.3–7.4)	5.8 (4.6–6.9)	5.8 (4.2–7.8)	0.941
Uric acid, *μ*mol/L	273.9 ± 103.9	281.6 ± 89.1	266.44 ± 116.2	0.230
Fibrinogen, g/L	6.0 ± 1.9	5.6 ± 1.82	6.4 ± 1.88	<0.001
Fasting glucose, mmol/L	8.5 (6.1–12.4)	7.9 (5.95–11.1)	8.9 (6.4–13.05)	0.063
eGFR	87 (62.4–100.3)	85.5 (62.0–99.4)	89.8 (65.4–102.5)	0.436
Albuminuria	151 (55.9)	71 (53.4)	80 (58.4)	0.407

Major amputation *n* (%)	41 (15.1)	14 (10.5)	27 (19.6)	0.038

Data are presented as either means ± standard deviation or median (interquartile range) for continuous variables or number (%) for categorical variables.

GNRI, geriatric nutritional risk index; BMI, body mass index; eGFR, estimated glomerular filtration rate; HDL-C, high-density lipoprotein cholesterol; LDL-C, low-density lipoprotein cholesterol.

**Table 2:** Independent predictor of all-cause mortality in DFU with amputations.

	Univariate analysis		Stepwise multivariate analysis	
	HR (95% CI)	*p* value	HR (95% CI)	*p* value
Age (years)	1,044 (1.021–1.068)	<0.001	1.046 (1.022–1.072)	<0.001
BMI	0.918 (0.850–0.991)	0.029		
Hypertension	1.867 (1.121–3.109)	0.016		
Hemoglobin, g/L	0.985 (0.972–0.998)	0.028		
Albumin, g/L	0.945 (0.906–0.985)	0.008		
Creatinine, *μ*mol/L	1.002 (1.001–1.003)	0.001		
eGFR	0.985 (0.977–0.992)	<0.001	0.987 (0.979–0.995)	0.002
GNRI	0.960 (0.938–0.983)	0.001	0.945 (0.921–0.971)	<0.001

HR, hazard ratio; CI, confidence interval. Other abbreviations are as in Table 1.

In the multivariate model, the following variables were added as independent variables: age, sex, history of hypertension, albuminuria hemoglobin level, estimated glomerular filtration rate, the presence of minor or major amputation, Wagner classification, and GNRI.

**Table 3:** Independent predictor of all-cause mortality in DFU with minor amputations.

	Univariate analysis		Stepwise multivariate analysis	
	HR (95% CI)	*p* value	HR (95% CI)	*p* value
Age (years)	1,053 (1.027–1.079)	<0.001	1.061 (1.033–1.089)	<0.001
BMI	0.918 (0.850–0.991)	0.029		
Cerebral vascular disease	1.979 (1.070–3.662)	0.016		
Coronary artery disease	2.286 (1.119–4.671)	0.023	2.291 (1.086–4.836)	0.030
Creatinine, *μ*mol/L	1.002 (1.001–1.004)	0.008		
eGFR	0.986 (0.977–0.994)	0.001		
GNRI	0.960 (0.935–0.987)	0.003	0.936 (0.908–0.965)	<0.001

HR, hazard ratio; CI, confidence interval. Other abbreviations are as in Table 1.

In the multivariate model, the following variables were added as independent variables: age, sex, hemoglobin, history of hypertension, cerebral vascular disease, coronary artery disease, albuminuria hemoglobin level, estimated glomerular filtration rate, Wagner classification, and GNRI.
